# Sex-related perception of body image, attitude toward food, and nutritional status of university students, and their relationship with physical activity level

**DOI:** 10.3389/fpsyg.2025.1567566

**Published:** 2025-04-22

**Authors:** Patricia Ruiz-Bravo, Sonia García-Merino, Bárbara Rodríguez-Rodríguez, Nuria Mendoza Laiz, Germán Díaz Ureña

**Affiliations:** Faculty of Health Sciences, Universidad Francisco de Vitoria, Madrid, Spain

**Keywords:** body image, attitude toward food, nutritional status, physical activity, university student

## Abstract

University life is a critical period for acquiring and consolidating healthy habits. This study examined the influence of sex on the body image perception, attitude toward food, nutritional status, and lifestyle habits of university students. This descriptive observational study included 163 university students from a program of Health Sciences. The data were collected with digitally distributed, self-administered questionnaires. Instruments such as the International Physical Activity Questionnaire were used to measure physical activity, the Eating Attitudes Test-26 to assess attitudes toward food, the Multidimensional Body Self Relations Questionnaire and Gardner’s assessment for body image perception, and the Prevención con Dieta Mediterránea for adherence to the Mediterranean diet. Body composition was evaluated with an InBody 770 device. Most of the values found were within the appropriate range and not significant in practice, except in the Gardner test for women, where relevant values were found. Female participants demonstrated greater dissatisfaction with their body image and higher EAT-26 scores, indicating an increased risk for eating disorders. Male participants exhibited higher body mass index and physical activity levels. No significant differences were observed in adherence to the Mediterranean diet between the sexes. Correlations between the variables revealed that body dissatisfaction was associated with a higher percentage of body fat and visceral fat area in both sexes. Gender differences in body image perception and attitude toward food underscore the need for sex-specific interventions. Promoting a positive body image perception and healthy eating habits is essential for improving the physical and mental health of university students. Educational programs should consider these differences and emphasize the promotion of physical activity and adherence to balanced diets.

## 1 Introduction

Adolescence is considered a critical stage in the acquisition of healthy habits and behaviors. During this period, patterns of eating, physical activity, and social relationships acquired throughout childhood are consolidated and provide the foundation for future behavior (World Health Organization, 2024). Upon completion of this stage, young individuals who elect to pursue higher education transition to adulthood in a highly stimulating environment characterized by numerous changes, wherein social life and academic demands present significant challenges. This process of transition to adulthood involves substantial changes, with the onset of adolescence occurring earlier and its conclusion later. Consequently, adolescence has been redefined as the period between 10 and 24 years of age, presenting new challenges to society (Sawyer et al., 2018).

This demanding environment can compromise the maintenance of healthy habits, particularly dietary ones. Research indicates that lack of time, stress related to academic responsibilities, and limited economic resources can impede the nutritional intake of young individuals (Deliens et al., 2014; Stok et al., 2018). The consumption of fast food and a poorly diversified diet, along with irregular eating patterns, such as omitting breakfast or engaging in frequent snacking, are characteristic of this population (Hilger et al., 2017).

This relationship with food is not merely a reflection of the demands imposed by university life. The social pressure to conform to a stereotypical body image promoted by social networks, which encourages thin physiques in women and muscular physiques in men, can have a significant impact on the mental and emotional well-being of young individuals (Alfano et al., 2011; Cho and Lee, 2013). In this context, two distinct realities can be observed: on the one hand, the percentage of young people who are overweight or obese, and on the other hand, those who are underweight. In both instances, pathological behaviors may be associated with eating disorders (EDs) (Sepulveda et al., 2010). While body composition is an important health indicator, the primary focus should be on establishing healthy behavioral patterns.

Body dissatisfaction and EDs are closely associated. Sepulveda et al. (2010) observed that older students with high self-esteem had low scores on the Eating Disorder Inventory. Specifically, in women, depression, the paranoid dimension, diet, and body dissatisfaction were associated with unhealthy eating patterns, whereas in men, diet, body dissatisfaction, and interpersonal sensitivity were key aspects. The results confirmed that unstructured eating habits tend to affect specific vulnerable groups.

However, a positive correlation was observed between participation in sport activities and body image perception, which underlines the importance of sport in improving self-awareness, well-being and health. Participation in sport activities can improve feelings of self-efficacy, self-determination and self-acceptance. The pursuit of positive outcomes can motivate individuals to initiate and maintain regular exercise regimes (Toselli and Spiga, 2017).

Given this disparity, targeted health education programs for students should focus on altering perceptions of body size and weight, emphasizing the importance of a healthy lifestyle that incorporates sports participation and promotes a realistic and healthy body shape to counteract media influence, particularly among women (Toselli and Spiga, 2017).

Furthermore, physical exercise can improve body self-esteem, especially in terms of physical fitness and physical strength (Zhang et al., 2024) and a positive perception of body image is associated with healthier dietary habits, more frequent physical activity and better psychological health outcomes (Jiménez-Morcillo et al., 2024). However, the pursuit of an idealized body image may lead to less healthy exercise patterns and behaviors than intended, the complex relationship between exercise and eating disorders requires tailored interventions to address maladaptative exercise behaviors (Liao et al., 2024).

Young individuals and/or adolescents frequently conceptualize health in relation to their body size or shape, and these culturally determined expectations about the body, especially for women, often impede their engagement in healthy behaviors, including physical activity (Witmer et al., 2011).

Various studies have investigated the relationship between body image perception and health behaviors among university students. With a sample of 600 university students, Elipe-Miravet et al. (2020) examined the associations between dietary care, body image perception, and body satisfaction as well as their potential impact on perceived mental health. The findings indicated that young individuals who maintained proper dietary habits exhibited high levels of body satisfaction and reported better mental health outcomes.

The impact of dietary habits on both physical and psychological well-being is well established. The Mediterranean diet has garnered substantial support from the scientific community. Numerous studies have demonstrated the beneficial effects of this dietary pattern on life expectancy and quality of life, as it has been shown to positively influence health by reducing the risk of cardiovascular disease, Alzheimer’s disease, depression, diabetes, and cancer. In this context, specifically regarding university students, Zurita-Ortega et al. (2018) observed that greater adherence to the Mediterranean diet was associated with an enhanced physical self-concept and increased levels of physical activity.

Physical self-concept is closely related to body image. In the study conducted by Baceviciene et al. (2020), the authors observed that a more positive body image was positively associated with the domains of psychological and physical quality of life in both female and male students. In addition, disordered eating was associated with diminished psychological domains in women. Positive body image traits (satisfaction with body areas) appeared to play a significant role in the quality of life of both sexes.

Body dissatisfaction in young individuals can be a precursor to the development of disordered and/or pathological eating behaviors. Therefore, it is crucial to analyze how to help young people navigate a social environment in which unrealistic and potentially unhealthy body ideals exist. Understanding the relationship between lifestyle factors such as diet, exercise, and body image satisfaction will help facilitate the design of educational programs to prevent physical and mental health issues, ultimately fostering more balanced and psychologically healthy individuals.

Furthermore, although an increasing number of studies are being conducted to analyze the differences between men and women and their perception and/or satisfaction with their body image, the vast majority have exclusively addressed the female sex partly because they are at a higher risk of EDs and lower body image satisfaction. Therefore, based on the aforementioned considerations, and with the aim of filling the gap in the literature in this area, the purpose the objective of this study was to analyze the influence of sex on the perception of and/or satisfaction with body image in university students of a health sciences degree and its relationship with nutritional status and lifestyle habits, such as physical activity, adherence to the Mediterranean diet, and attitude toward food, through validated instruments, to add reliability to the measurement of variables.

## 2 Materials and methods

### 2.1 Study design

This study employed a descriptive observational design. Recruitment and data collection were completed by 2024. The data were obtained through self-administered questionnaires distributed digitally, utilizing the Microsoft Forms application (Version 365) and disseminated through an institutional digital platform (CANVAS). This study was conducted in accordance with the STROBE guidelines for observational epidemiological studies (von Elm et al., 2008).

### 2.2 Procedure

The questionnaires were administered over a period of 4 weeks, commencing with “Week 0,” during which the IPAQ was administered to assess the participants’ physical activity level. Subsequently, in “Week 1,” participants completed questionnaires focusing on nutritional aspects (EAT-26 and PREDIMED). During “Week 2,” questionnaires related to Body Image Perception (Multidimensional Body-Self Relations Questionnaire [MBSRQ] and Gardner’s Body Image Assessment) were administered. Finally, in “Week 3,” the body composition test was conducted with InBody Model 770.

### 2.3 Participants

The inclusion criteria were enrollment in one of the degrees offered by the Faculty of Health Sciences at Universidad Francisco de Vitoria, such as Physical Activity and Sports Sciences (CAFyD), Nutrition, Nursing, and Physiotherapy, and the participants had to be aged between 18 and 25 years. Excluded were those who did not complete all required tests and/or did not provide informed consent.

An initial sample size of 128 was estimated, assuming an effect size of *d* = 0.5, *α* error = 0.05, statistical power of 1 - *β* = 0.8, and a group ratio of 1. Anticipating 10% sample attrition and ensuring an *a priori* estimated sample size, 140 participants were recruited. A medium effect size was assumed based on the differences between men and women. These differences were anticipated to be present but not substantial. The sample size was determined using standard power analysis techniques, ensuring sufficient power to detect medium-sized effects with a high degree of confidence.

### 2.4 Variables

The variables investigated in this study included:

1. Body image perception.
2. Attitude toward food.
3. Nutritional status: body mass index (BMI), percentage of body fat (PBF), and visceral fat area (VFA).
4. Adherence to the Mediterranean diet.
5. Physical activity level.

### 2.5 Data sources/measures

To control for response bias, it was stated that the questionnaires were anonymous, confidentiality was assured, and there were no right or wrong answers when administering them.

#### 2.5.1 Tests used to evaluate body image perception (MBSRQ and Gardner’s assessment)

The MBSRQ (Cash, 2000) was used to assess the attitudinal, cognitive, and behavioral aspects of the body image construct. The Spanish translation developed by Botella et al. (2009) was used in this study. All items are rated on a Likert scale with five response options. These options vary according to the items: items 1–57, ranging from Strongly Disagree to Strongly Agree; item 58, from Never to Very Often; items 59 and 60, from Very Underweight to Obese; and 61 to 69, from Very Dissatisfied to Very Satisfied. For interpretation, higher scores indicate greater satisfaction.

The Body Image Assessment (Gardner et al., 1999) comprises two contour scales that use frontal views of adult men and women of median height and weight in the United States. The scale consists of 13 silhouettes, 8 cm in height, without any attributes such as hair and face. Thirteen figures were presented in this study. Participants were required to identify with one of them and subsequently select the one they desired to have or identify with. The results were interpreted by subtracting the image they perceived themselves from the image they wanted to have. A value greater than zero indicated dissatisfaction with the desire to be thinner. If it was less than zero, it was associated with dissatisfaction and the desire to have a larger body volume. If the result was zero, it was interpreted as satisfaction. This methodology allowed for the determination of a body image satisfaction index.

#### 2.5.2 Tests used to evaluate attitudes toward food (EAT-26)

The EAT-26 questionnaire was used to identify symptoms and concerns related to fear of gaining weight, tendency to lose weight, and the presence of behaviors related to restrictive eating patterns. The original questionnaire developed by Garner and Garfinkel (1979) consisted of 40 questions, and a short version of 26 questions was later developed (Grande et al., 2003). This study employed the Spanish version validated by Rivas et al. (2010). The items are presented on a Likert scale with six response options. The total score was obtained by coding the scores as follows: Scores from 1 to 3 were coded as 0, 4 as 1, 5 as 2, and 6 as 3, respectively. The only exception was item 25, whose responses were inversely scored. The cutoff point for differentiating between asymptomatic and symptomatic subjects was 19 points. Subjects with scores below 19 points were classified as not at risk of an ED, and those with scores equal to or greater than 20 as at high risk of an ED.

#### 2.5.3 Tool to evaluate nutritional status

BMI, PBF, VFA, and the InBody Model 770 were used. Initially, data from each participant, including sex, age, and height, were recorded on a screen with Microsoft Excel (Version 365). Height was measured with a stadiometer (SECA 700) following the International Society for the Advancement of Kinanthropometry (2019) protocol, and participants were instructed to remove their footwear and inhale deeply during the measurement. Weight and body composition data were obtained through direct segmental multifrequency bioelectrical impedance analysis, using an InBody 770 scale previously sanitized with alcohol. The measurement prerequisites included refraining from moderate-intensity physical activity in the previous 24 h, urinating at least 30 min prior, being barefoot, not wearing metal accessories, fasting for at least 4 h before, and confirming adherence to these requirements before measurement. The device employs tetrapolar tactile electrodes with eight contact points (two on each hand and foot) which perform impedance measurements at six different frequencies (1, 5, 50, 250, 500, and 1,000 kHz) for each body segment. The skin of the plantar surfaces and palms was cleaned with an electroconductive wipe before the participants positioned themselves on the tactile electrodes of the platform, maintaining an orthostatic position, and holding the equipment handles with lateral abduction of the arms at approximately 20° and flexion of the scapulohumeral joint at approximately 30°, ensuring contact with the eight points of the tactile electrodes. The legs, thighs, arms, and trunk were not in contact before activating the equipment, which induced an electric current through the body of the participant, thereby estimating various body composition indicators (Guedes et al., 2019).

#### 2.5.4 Questionnaire to evaluate adherence to the Mediterranean diet

The PREDIMED questionnaire was used to evaluate adherence to the Mediterranean diet (Martinez-Gonzalez et al., 2012), as scientific evidence suggests that greater adherence to the diet is associated with improved physical and mental health outcomes (Schröder et al., 2011). This questionnaire includes 14 questions that assess the number of servings and frequency of consumption of typical Mediterranean diet foods, such as olive oil, nuts, fruit, wine, seafood, and legumes, as well as the low consumption of non-traditional foods, like red or processed meats, sugary beverages, desserts, and confectionery. Each point on the questionnaire indicates greater compliance with the Mediterranean diet, with scores approaching 14 reflecting a high level of adherence, scores between 8 and 11 indicating a medium level, scores between 5 and 7 reflecting a low level, and scores of 5 or less reflecting very low adherence.

#### 2.5.5 Assessment of physical activity level with the IPAQ

Physical activity was assessed with the IPAQ in its short form (Carrera, 2017), which comprises seven items and provides data on the duration of walking, moderate-intensity activities, vigorous-intensity activities, and sedentary behaviors over the preceding 7 days, encompassing four domains of physical activity: leisure time, household maintenance, occupational, and transportation. This questionnaire, recommended for adults aged 18–69 years (Mantilla-Toloza and Gómez-Conesa, 2007), has been validated in 12 countries (Ruiz et al., 2012). For the analysis, all obtained metabolic expenditures were aggregated and expressed in METs/week, with vigorous activity multiplied by 8 METs, moderate activity by 4 METs, and low-intensity activity by 3.3 METs.

### 2.6 Data analysis

Descriptive statistical analyses were conducted for all variables, including means, standard deviations, and 95% confidence intervals. Comparisons of means between two independent groups were performed with the t-test for independent samples, with a two-tailed *p*-value used for testing the equality of means. The effect sizes for the mean differences were calculated with Cohen’s d and interpreted according to Cohen (1988) (small, 0.2; medium, 0.5; large, 0.8). Pearson’s correlation coefficients were calculated for each group to assess the relationships between variables. Correlations were considered moderate if the coefficient exceeded 0.4 and high if it exceeded 0.6. For the confidence intervals of the correlations, a bias adjustment was applied to ensure accurate estimation. Subsequently, the correlations between the two groups were compared with Fisher’s Z transformation to evaluate whether the correlations were significantly different between the groups Effect size index (*q*) to compare these differences were calculated and interpreted according to Cohen (1988) (small, 0.1; medium, 0.3; large, 0.5). The reliability and validity of the EAT26 and MBSRQ questionnaires were evaluated using Kaiser-Meyer-Olkin (KMO), Bartlett’s test of sphericity, Cronbach’s alpha and McDonald’s Omega. All statistical analyses were performed in Rstudio software (Version 2024.09.1 + 394). Packages ggstatsplot (Patil, 2021), epiR (Version 2.0.76), and Psych (Revelle, 2024) were used to perform calculations and generate figures. A *p*-value of *p* < 0.05 was established for all comparisons.

## 3 Results

Of 181 recruited participants, 163 met the inclusion criteria (96 women and 67 men). Eighteen participants were excluded because they failed to meet the specified age requirement for participation in this study.

The EAT26 questionnaire demonstrated acceptable sampling adequacy with a Kaiser-Meyer-Olkin (KMO) value of 0.77, and Bartlett’s test of sphericity was significant (*p* < 0.001). This questionnaire showed a Cronbach’s alpha of 0.82 and a McDonald’s Omega of 0.87. The MBSRQ had a KMO value of 0.76, and Bartlett’s test was significant (*p* < 0.001), with a Cronbach’s alpha of 0.91 and a McDonald’s Omega of 0.94.

**Figure 1:**
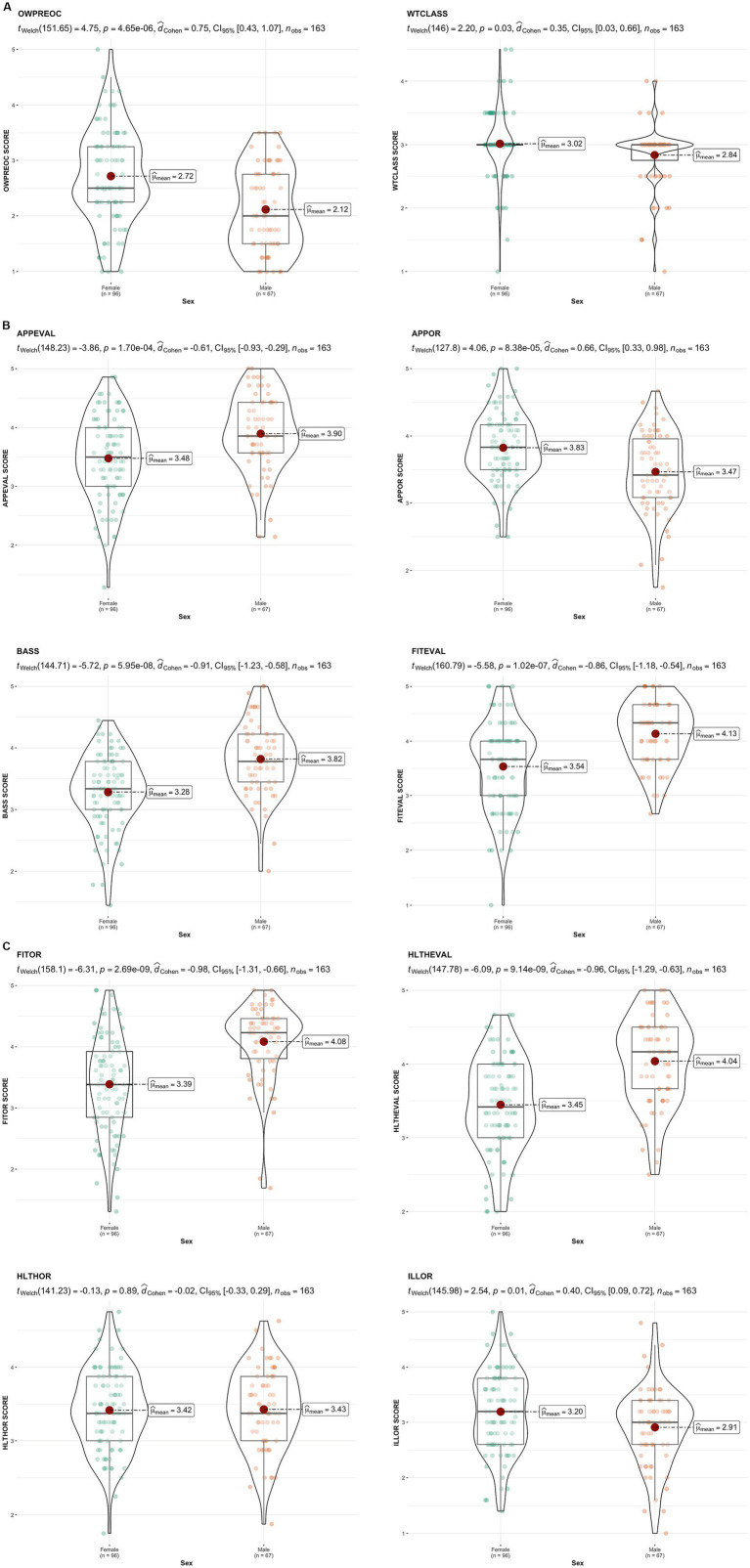
**(A–C)** MBSRQ. APPEVAL, appearance evaluation; APPOR, appearance orientation; FITEVAL, fitness evaluation; FITOR, fitness orientation; HTLHEVAL, health evaluation; HTLHOR, health orientation; ILLOR, illness orientation; BASS, body area satisfaction; OWPREOC, overweight preoccupation; WTCLASS, self-classified weight.

Figure 1 presents the descriptive values and group comparisons of the MBSRQ. With the exception of “health orientation,” all other variables exhibited significant differences between men and women, with medium and large effect sizes.

**Figure 2:**
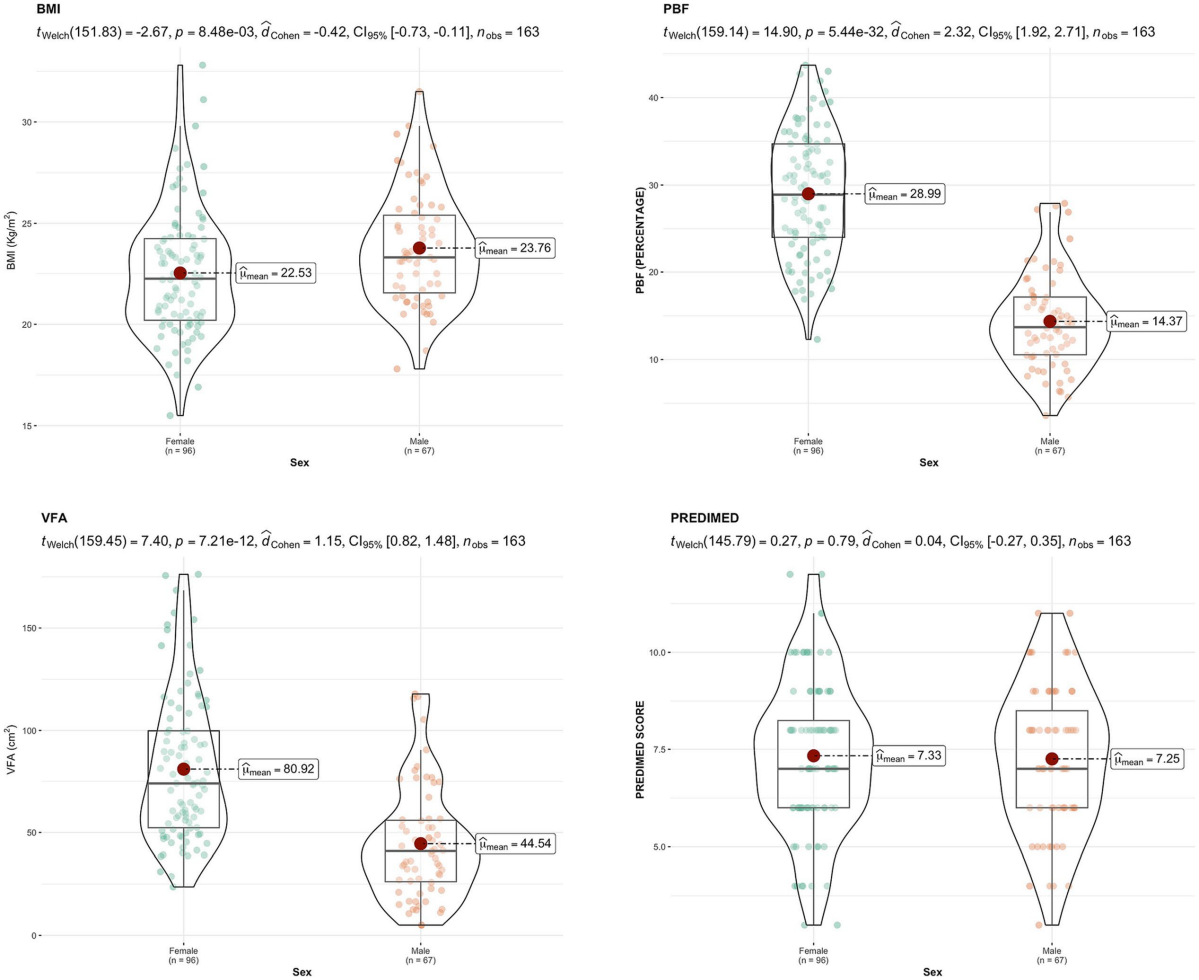
Nutritional status and PREDIMED. BMI, body mass index; PBF, percentage of body fat; VFA, visceral fat area.

Next, Figure 2 presents the descriptive values and group comparisons of nutritional status and PREDIMED scores.

**Figure 3:**
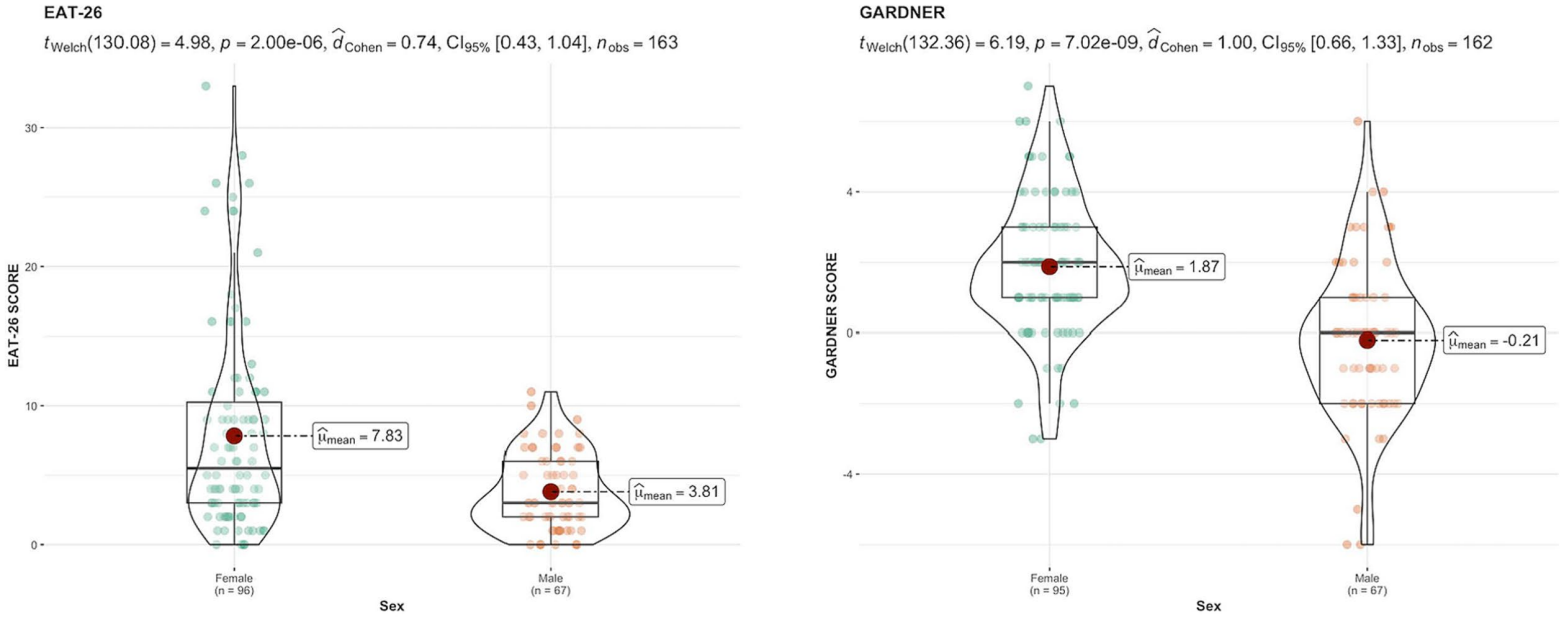
EAT-26 and Gardner’s assessment.

Figure 3 illustrates the values obtained from the EAT-26 and Gardner’s assessment. Statistically significant differences were observed between the male and female participants in both groups, with a large effect size.

Next, Table 1 presents the moderate, high, and very high correlations (*r* > 0.4) between the men and women. In addition, a comparison of the two correlations is provided. With the exception of “GARDNER – Appearance Evaluation,” “GARDNER – Body Areas Satisfaction,” “Fitness Orientation – Health Evaluation,” and “Health Evaluation – Health Orientation,” no significant differences existed between the correlations calculated for women and those calculated for men.

**Table 1:** Correlations between different variables.

	Female			Male					
Variables		CI (95%)			CI (95%)		*z*-value	*p*-value	*q*-value
	*r*	LL	UL	*r*	LL	UL			
VFA – BMI	0.86†	0.91	0.96	0.95†	0.92	0.97	−0.81	0.42	0.11
VFA – PBF	0.94†	0.80	0.91	0.83†	0.74	0.89	0.70	0.48	0.13
VFA – GARDNER	0.49†	0.32	0.63	0.61†	0.43	0.74	−1.04	0.30	0.17
VFA – OWPREOC	0.46†	0.29	0.61	0.48†	0.27	0.65	−0.14	0.89	0.02
VFA – WTCLASS	0.57†	0.42	0.69	0.51†	0.31	0.67	0.50	0.62	0.08
PBF – BMI	0.76†	0.66	0.83	0.77†	0.65	0.85	−0.22	0.82	0.04
PBF – GARDNER	0.43†	0.25	0.58	0.59†	0.41	0.73	−1.37	0.17	0.22
PBF – OWPREOC	0.43†	0.25	0.58	0.44†	0.22	0.61	−0.11	0.92	0.02
PBF – WTCLASS	0.52†	0.36	0.65	0.50†	0.29	0.66	0.19	0.85	0.03
BMI – GARDNER	0.53†	0.36	0.66	0.69†	0.53	0.80	−1.61	0.11	0.26
BMI – OWPREOC	0.43†	0.25	0.58	0.49†	0.28	0.65	−0.47	0.64	0.08
BMI – WTCLASS	0.67†	0.54	0.76	0.64†	0.47	0.76	0.30	0.76	0.05
EAT26 – OWPREOC	0.50†	0.33	0.63	0.46†	0.25	0.63	0.29	0.77	0.05
GARDNER – APPEVAL	−0.47†	−0.61	−0.30	−0.16	−0.39	0.08	−2.17	0.03*	0.35
GARDNER – BASS	−0.46†	−0.60	−0.28	0.02	−0.23	0.26	−3.14	>0.01†	0.51
GARDNER – OWPREOC	0.51†	0.34	0.64	0.48†	0.27	0.64	0.26	0.79	0.04
GARDNER – WTCLASS	0.60†	0.46	0.72	0.61†	0.43	0.74	−0.08	0.94	0.01
APPEVAL – HTLHEVAL	0.40†	0.22	0.56	0.43†	0.21	0.60	−0.19	0.85	0.03
APPEVAL – BASS	0.68†	0.56	0.78	0.63†	0.45	0.75	0.61	0.54	0.10
APPEVAL – OWPREOC	−0.31†	−0.48	−0.11	−0.41†	−0.59	−0.18	0.71	0.48	0.11
FITEVAL – FITOR	0.58†	0.42	0.69	0.39†	0.16	0.58	1.49	0.14	0.24
FITOR – HTLHEVAL	0.30†	0.11	0.48	0.57†	0.38	0.71	−2.05	0.04*	0.33
FITOR – HLTHOR	0.43†	0.25	0.58	0.59†	0.41	0.73	−1.35	0.18	0.22
HTLHEVAL – HLTHOR	0.15	−0.06	0.34	0.44†	0.22	0.61	−1.98	0.047*	0.32

LL, lower limit; UL, upper limit; APPEVAL, appearance evaluation; APPOR, appearance orientation; FITEVAL, fitness evaluation; FITOR, fitness orientation; HTLHEVAL, health evaluation; HTLHOR, health orientation; ILLOR, illness orientation; BASS, body area satisfaction; OWPREOC, overweight preoccupation; WTCLASS, self-classified weight; BMI, body mass index; PBF, percentage of body fat; VFA, visceral fat area; *q*-value, effect size index.

* *p* < 0.05.

† *p* < 0.01.

In the two correlations involving GARDNER (“GARDNER – Appearance Evaluation” and “GARDNER – Body Areas Satisfaction”), a significant correlation was observed in women, while no significant correlation was found for the men. Regarding the correlation “Fitness Orientation – Health Evaluation,” although a significant correlation was present in both men and women, it was significantly higher in men. Only in the correlation “Health Evaluation – Health Orientation” was there no significant correlation in women, whereas a significant correlation was observed in men. In all the correlations mentioned there was a medium effect size.

## 4 Discussion

The present study investigated the body image perception, attitude toward food, and nutritional status of university students, as well as their relationship with physical activity levels. The findings demonstrated statistically significant differences in all analyzed variables based on sex, with the exception of the PREDIMED questionnaire score and health orientation factor of the MBSRQ. Specifically, women exhibited significantly higher scores than men on the EAT-26, indicating a higher risk of EDs in terms of attitudes and behaviors related to food and body weight. Furthermore, the BMI was higher in men compared to women. However, the PBF was higher in the women.

However, most of the values found were within the appropriate range for sex and age. Specifically, in the case of the EAT26 the values were below 20, which makes it not clinically relevant. On the other hand, in the perception of body image, in the MBSRQ the participants obtained adequate values. Instead, in the Gardner test, women showed relevant values to be considered in practice. Finally, in terms of IPAQ PREDIMED and nutritional status, the values are in appropriate ranges.

### 4.1 Body image perception (MBSRQ and Gardner’s assessment)

In general, women exhibit a higher level of attention to their body image (APPOR) but demonstrate lower satisfaction with it (APPEVAL) and various body areas (BASS) than men. This phenomenon may be associated with the findings of Pritchard et al. (2021) and Toselli and Spiga (2017), who observed that social–physical anxiety and the desire to be thinner are more significant in women, potentially due to societal standards being more flexible for men.

Regarding health evaluation (HTLHEVAL), men scored significantly higher than women (4.04 vs. 3.45), indicating a perception of better health. These results diverge from those reported by Ramos-Jiménez et al. (2017), who found no significant differences in health perceptions between the sexes. However, in terms of health orientation (HTLHOR), no significant differences were observed, contradicting Zanon et al. (2016), who suggested that women are more concerned about their health and physical appearance. Despite similar values in health orientation, men engaged in more activities to maintain it, as reflected in physical activity levels (IPAQ). Women perceived themselves as having a poorer physical condition (FITEVAL), devoted less attention to improving it (FITOR), exhibited greater concern about being overweight (OWPREOC), and evaluated themselves more critically (WTCLASS), aligning with Garner et al. (1982).

In terms of body image perception, significant differences were observed between sexes. Women perceived themselves as heavier and desired to be thinner, while men desired a larger body volume. These findings are consistent with Toselli and Spiga (2017), who found that women perceived themselves as slightly overweight and desired to be thinner, whereas men desired to be larger.

Regarding body dissatisfaction, this study revealed that women exhibited higher levels of dissatisfaction with their body image than men. This finding aligns with Duarte et al. (2021) and Boutahar et al. (2019), who noted a high prevalence of distorted self-perception and significant body image dissatisfaction among women.

Women also tended to overestimate their body size, consistent with Laus et al. (2015), and were more susceptible to external influences regarding body image, as noted by You and Shin (2020) and Zanon et al. (2016). Discrepancies between current and ideal body image were associated with greater body concerns and lower satisfaction, particularly among women, as found by Novella et al. (2015). This finding is congruent with the results of the present study, wherein discrepancies between current and ideal body image perceptions were correlated with increased dissatisfaction and concern about weight. These differences in the perception and satisfaction with body image between men and women may be influenced by various cognitive biases. Body image perception is influenced by several biases, such as confirmation bias, social comparison, attention, and memory. These could lead to selective interpretations and memories that affect the perception of one’s own body image (Barrios, 2025; Borda Pérez et al., 2016; Inzunza Rosales et al., 2023). Furthermore, this data may be conditioned by the participants’ responses regarding body image perception, tending to give answers that are considered more socially acceptable than their true opinions or behaviors (social desirability). (Júnior and Patrício, 2022). For example, comparisons with social ideals and attention to negative aspects of the body can increase dissatisfaction and concern about weight.

### 4.2 Attitudes toward food

Regarding attitudes toward food evaluated with the EAT-26, significant differences were observed between the scores obtained by men and women. Women exhibited a higher mean EAT-26 score than men, suggesting an elevated risk of potential EDs. This finding aligns with previous research that identified gender disparities in attitudes toward food and EDs. For instance, Aiello et al. (2022) assessed the prevalence of orthorexia nervosa and eating attitudes among Italian and Spanish university students, using the EAT-26 questionnaire. The results demonstrated that although both men and women exhibited high scores on the EAT-26, it was more pronounced in women, with a higher prevalence of orthorexia nervosa in the Spanish sample. Similarly, consistent with our study, women tended to be at a higher risk of an ED than men, suggesting that irrespective of cultural context, women may be more susceptible to developing eating-related attitudes and behaviors, as also observed in other cultural contexts (Martínez-Munguía et al., 2015).

Moreover, Chin et al. (2020) found that satisfaction with body size and body appreciation were significant factors in eating attitudes, which corresponds with our findings of a substantial correlation between the EAT-26 and overweight preoccupation (OWPREOC) in both sexes. In addition, Ohara et al. (2014) used the Dutch Eating Behavior Questionnaire (DEBQ) to evaluate the relationship between eating behavior and body image perception. The results indicated that women exhibited higher scores in restrained, emotional, and external eating than men, which is consistent with our finding of higher EAT-26 scores among women.

Additionally, Da Silva et al. (2018) found that concerns about body image perception and aspects of emotional eating significantly affect women’s quality of life, whereas general body appearance and cognitive restraint were more relevant for men. These findings are consistent with our results, indicating greater concern about food and being overweight among women, which can negatively influence body image perceptions. Furthermore, Heiman and Olenik-Shemesh (2019) further corroborate these findings, demonstrating that women report greater dissatisfaction with their body appearance and exhibit healthier eating habits than men.

### 4.3 Nutritional status

Regarding nutritional status, adherence to the Mediterranean diet (PREDIMED) showed no statistically significant differences between males and females, consistent with Sanz-Martín et al. (2023). Both sexes exhibited high adherence; however, our study showed lower values: 46.87% of females and 47.76% of males scored above 8 (moderate–high adherence), and only 4.17% of females and 2.99% of males scored 11 or higher (high adherence). In contrast, Zanon et al. (2016) reported 11.7% moderate to high adherence.

In terms of the association between adherence to the Mediterranean diet and other variables, no statistically significant differences were observed in our study. Specifically, no significant differences were identified between adherence to the Mediterranean diet and physical activity level, consistent with García-Pérez-de-Sevilla et al. (2021). Conversely, Romero-Blanco et al. (2022) and Alba (2019) found that higher physical activity levels correlated with greater adherence to the Mediterranean diet.

However, regarding body composition, statistically significant differences were observed in BMI, PBF, and VFA between males and females. Additionally, a significant correlation was noted between BMI and body image dissatisfaction, as measured with Gardner’s assessment. These findings are similar to those reported by Khalaf et al. (2021), who found a negative association between BMI and body appreciation, indicating that students with higher BMI tend to exhibit lower body appreciation. Although the authors did not observe significant differences in body appreciation between men and women, which contrasts with our results, wherein women demonstrated greater dissatisfaction.

Furthermore, Boutahar et al. (2019) reported a high prevalence of body image dissatisfaction, particularly among overweight and obese individuals. The authors also observed that body image dissatisfaction was comparable between men and women, which differed from our results. This discrepancy may be attributed to differences in evaluation methods and sample characteristics. Conversely, Zaccagni et al. (2020) found that their female participants exhibited greater dissatisfaction with their body image and a tendency to overestimate body fat, whereas men tended to underestimate it.

In our study, a significant correlation was observed between PBF and body image dissatisfaction, which is consistent with the findings of Duarte et al. (2021). They reported a high prevalence of body image dissatisfaction and distorted self-perception of body size. In contrast, Nakanishi et al. (2018) found that women tended to have an ideal body image with lower body weight, irrespective of their BMI. The authors also reported that a lack of exercise and irregular eating habits were common among students with a higher BMI, which could influence body image perception and dissatisfaction.

Similarly, Soto-Ruiz et al. (2015) found that a high percentage of students had a body image perception that did not correspond to reality, with women tending to overestimate or underestimate their BMI. This finding is consistent with our results: women exhibited greater body image dissatisfaction as their BMI and PBF increased. However, the authors did not observe a strong correlation between BMI and body image dissatisfaction, which differed from our findings.

Regarding eating habits, physical activity levels, and their relationship with body satisfaction, Hao et al. (2023) found that body dissatisfaction was higher in women and associated with greater muscle mass and emotional eating behaviors. The results also suggested that improving body satisfaction could positively affect students’ nutritional statuses and lifestyle habits. These results are comparable to ours: Women with higher PBF and VFA scores demonstrated greater body image dissatisfaction. In contrast, this study differed in that no correlations were observed between body image satisfaction and adherence to the Mediterranean diet or physical activity levels.

### 4.4 Physical activity

Physical activity was evaluated with an international physical activity questionnaire. The results demonstrated significant differences between men and women regarding the quantity of physical activity performed, with men reporting higher levels of physical activity than women. These data are consistent with those reported by Zanon et al. (2016), wherein a sample of young Italian athletes exhibited that men reported greater participation in vigorous physical activities compared to women. This pattern was replicated in our study, where men demonstrated a greater propensity to engage in intense physical activity. Furthermore, Zanon et al. (2016) emphasized that physical activity was positively associated with enhanced body image perception, which aligns with Hao et al. (2023) and our findings, indicating a significant correlation between physical activity and body image satisfaction, particularly in men.

Morever, Toselli and Spiga (2017) reported that males engaged in more sports activities than females, and those with reduced sports participation exhibited greater body dissatisfaction. This finding aligns with our observations. Lower physical activity was associated with increased body dissatisfaction, particularly among females.

Similarly, Ramos-Jiménez et al. (2017) determined that body image satisfaction was a reliable indicator of participation in sports. In our study, although body image satisfaction was not directly evaluated as a predictor of physical activity, a significant correlation was observed between physical activity and body image satisfaction, suggesting that individuals with a more positive perception of their bodies tend to be more physically active.

Furthermore, More and Phillips (2019) found that body dissatisfaction was related to a lower frequency of both cardiovascular and strength physical activities due to reduced levels of intrinsic regulation. This study also highlighted that in females, body dissatisfaction was partially mediated by controlled regulations, suggesting that females dissatisfied with their bodies may engage in exercise more frequently for external reasons, such as social pressure or seeking approval.

Finally, Aiello et al. (2022), who also utilized the IPAQ to evaluate physical activity, found significant sex differences, with males reporting higher levels of intense physical activity. This finding corroborates our results and reinforces the need to consider sex differences when designing interventions to promote physical activity.

## 5 Practical applications

Our findings have significant practical implications for the design of health interventions and programs that target university students. First, the high prevalence of body image dissatisfaction emphasizes the necessity of developing specific programs that address these concerns and promote a positive body image, taking into account gender differences, and aiming to enhance self-esteem and self-acceptance. Regarding attitudes toward food, the observed gender differences indicate the need for interventions that both incorporate educational and psychological support components, focus on promoting healthy eating habits, and address cultural, psychological, and contextual factors that influence body image perception and eating behaviors. Concerning nutritional status, interventions should consider BMI, PBF, and VFA as key indicators to improve body image perception and nutritional status, integrate regular evaluations of these indicators, and provide individualized feedback. Finally, gender differences in physical activity highlight the importance of designing exercise programs that are appealing and accessible to both sexes, promoting participation in moderate- and vigorous-intensity physical activities adapted to individual preferences and needs as a strategy to improve body image perception and reduce body dissatisfaction. Overall, our findings underscore the importance of considering gender in the development of public health strategies and intervention programs aimed at improving body image perception and health-related behaviors in university populations.

### 5.1 Limitations and recommendations for future research

This study presents certain limitations that should be considered when interpreting the findings. First, the use of self-reported questionnaires for data collection may introduce biases related to participants’ subjectivity, recall inaccuracies, and social desirability effects. Although validated instruments such as the IPAQ, PREDIMED, MBSRQ, Gardner’s assessment, and the EAT-26 were employed, it is important to acknowledge that the inclusion of additional complementary tools could have provided a more precise and comprehensive assessment of the analyzed variables. In particular, physical activity measurement through the IPAQ relies on participants’ ability to accurately recall and report their activity levels, which may not always align with objective data. Similarly, while the EAT-26 can identify risk symptoms related to eating disorders, it does not replace a clinical evaluation.

Another significant limitation is the cross-sectional design of this study, which prevents the establishment of causal relationships between the examined variables. For instance, although a correlation between physical activity and body image satisfaction was observed, it is not possible to determine whether engaging in physical activity enhances body image perception or whether individuals with a more positive body image perception are more likely to participate in physical activity. Future research should employ longitudinal designs to track these relationships over time and establish more robust causal patterns.

Regarding body composition assessment, bioelectrical impedance analysis (BIA) was conducted using the InBody 770 device. While this method provides a practical and non-invasive means of estimating body composition, it has certain accuracy limitations compared to dual-energy X-ray absorptiometry (DXA).

Sample size is another potential limitation, as it directly impacts the precision and generalizability of the results. A small sample increases the risk of bias and limits the extrapolation of findings to broader populations. Additionally, the sample distribution by sex was not balanced, which may have influenced the robustness of statistical comparisons. Future research should strive for a more equitable representation of males and females and include different BMI categories to improve the applicability of the findings.

To address these limitations, future studies should adopt more rigorous methodological approaches, such as mixed-method designs that integrate self-reported data with objective measurements and qualitative techniques. The implementation of complementary questionnaires that assess the same variables from different perspectives would allow for data triangulation, offering a more comprehensive understanding of the studied phenomena. Furthermore, given the observed differences in motivations for physical activity based on sex, further research should explore these aspects in greater depth to gain a more nuanced understanding of the factors influencing body image perception and engagement in physical activity across diverse populations.

Finally, considering the growing impact of digital and social media on body image perceptions, future studies should investigate their role in shaping attitudes and behaviors related to body perception and health habits. Additionally, exploring cultural, social, and environmental factors could facilitate the development of more effective strategies for promoting positive body image perception and psychological well-being in various contexts.

## 6 Conclusion

With this study we evaluated the influence of gender on body image perception and/or dissatisfaction in university students, as well as its relationship with attitudes toward food, nutritional status, and physical activity. The investigation revealed a high prevalence of body image dissatisfaction among university students. Female participants demonstrated lower scores on appearance-related aspects and exhibited greater attention to their body image and potential symptoms of illness. These sex-specific differences underscore the importance of considering sex in the design of health interventions and programs aimed at improving body image perception and health-related behaviors.

Regarding attitudes toward food, significant gender differences were observed in food-related concerns and ED behaviors. Female participants exhibited a higher risk of developing behaviors associated with these disorders in addition to more restricted and emotional eating patterns.

Regarding nutritional status, as evaluated with the PREDIMED and InBody tests, we observed that body image dissatisfaction in female participants increased with VFA and PBF. In both sexes, improving body satisfaction may have a positive impact on nutritional status. These findings suggest the importance of considering BMI, PBF, and VFA when designing interventions to improve body image perception and nutritional status in university students.

Conversely, the male participants reported higher levels of physical activity than the female participants and demonstrated a tendency to engage in a greater number of vigorous activities. However, no significant correlation was observed between physical activity level and body image dissatisfaction. These gender-based disparities in motivation for physical activity warrant further investigation in future research to acquire a more comprehensive understanding of the factors influencing physical activity and body image perception across diverse populations.

Consequently, female participants tended to exhibit greater body dissatisfaction and weight concerns, whereas male participants demonstrated a greater orientation toward physical condition and body volume. Although most of the values obtained were within appropriate ranges and not significant in practice, women showed relevant values in the Gardner test that should be considered. These findings emphasize the significance of considering sex as a variable in the design of health interventions and programs aimed at improving body image perception and health-related behaviors.

## Data Availability

The raw data supporting the conclusions of this article will be made available by the authors, without undue reservation.
